# Adiposity, cardiovascular, and health-related quality of life indicators and the reallocation of waking movement behaviors in preschool children with overweight and obesity: An isotemporal data analysis

**DOI:** 10.1371/journal.pone.0242088

**Published:** 2020-11-10

**Authors:** Erin E. Dooley, Kelley Pettee Gabriel, Harold W. Kohl, Casey P. Durand, Deanna M. Hoelscher, Courtney E. Byrd-Williams

**Affiliations:** 1 Risk Factor Assessment Branch, Epidemiology and Genomics Research Program, National Cancer Institute, Rockville, Maryland, United States of America; 2 Michael & Susan Dell Center for Healthy Living, The University of Texas Health Science Center (UTHealth) School of Public Health in Austin, Austin, Texas, United States of America; 3 Department of Epidemiology, School of Public Health, The University of Alabama at Birmingham School of Public Health, Birmingham, Alabama, United States of America; 4 Department of Kinesiology and Health Education, The University of Texas at Austin, Austin, Texas, United States of America; 5 Health Promotion and Behavioral Sciences, The University of Texas Health Science Center at Houston (UTHealth) School of Public Health, Houston, Texas, United States of America; University of New Brunswick, CANADA

## Abstract

**Background:**

Isotemporal substitution evaluates hypothetical time replacement scenarios of physical movement on health, with few studies conducted among ethnically diverse preschool-aged populations. This study examines the reallocation of waking movement behaviors on adiposity, cardiovascular, and quality of life indicators among low-income, majority Hispanic preschool-aged youth (2–5 years) with overweight.

**Methods:**

Participants wore an ActiGraph monitor (waist) and completed adiposity, cardiovascular, and health-related quality of life health assessments. Covariates included age, sex, ethnicity, and socioeconomic status. The isotemporal substitution approach was employed to address study aims.

**Results:**

Complete data were available for 131 preschoolers. For boys, reallocating 5 minutes of stationary time with light intensity, moderate to vigorous intensity, or total physical activity showed a relation with beneficial reductions in adiposity indicators; for girls, these relations were statistically null. For boys and girls, reallocating 5 minutes of stationary time [-2.2 (95% CI: -3.7, -0.7) mmHg], light intensity [-2.1 (95% CI: -3.7, -0.7) mmHg], or moderate intensity activity [-2.7 (95% CI: -5.0, -0.4) mmHg] to vigorous intensity activity was related to favorable systolic blood pressure. Reallocating 5 minutes of stationary time to moderate to vigorous intensity activity [0.6 (95% CI: -1.0, -0.1) mmHg] or total physical activity [-0.2 (95% CI: -0.3, -0.01) mmHg] was related to lowered systolic blood pressure. Reallocating 5 minutes of stationary time to moderate to vigorous intensity activity [0.6 (95% CI: -1.1, -0.02) bpm] was related to lowered resting heart rate. No significant results for quality of life were found.

**Conclusion:**

Reallocation of time from stationary time to other movement behaviors is associated with several favorable adiposity and cardiovascular health outcomes among preschool children with overweight and obesity.

## Introduction

The preschool years (ages 3–5 years) are a critical time for psychological and physiological development, including the development of adiposity throughout the life course [1–3]. Obesity trajectories have been found to start by age three [4] and become established by age five [5]. Comorbidities are also a concern as overweight and obesity is associated with elevated blood pressure (BP) [6–9] and reduced health-related quality of life (HRQoL) in preschool-age children [10, 11]. With half of childhood obesity prevalence occurring among children who have overweight during their preschool years [5], this age is a particularly critical time for development of excess adiposity.

Whole-day guidelines for young children suggest during waking periods children should be physically active throughout the day for health benefits [12–15]. With the rising childhood obesity prevalence in the U.S. [16, 17] and globally [18], there is an increasing need to examine how physical activity and sedentary behavior relate to health outcomes in children. The isotemporal substitution approach provides an opportunity to evaluate the relation of hypothetical time replacement scenarios across movement behaviors on health, while taking into account the other movement behaviors that occur within the day [19] and has been examined in relation to a variety of health outcomes–mental health, adiposity, fitness, and cardiometabolic biomarkers [20–22]. It has been found that substituting five minutes per day of sedentary, light, or moderate intensity activity with vigorous intensity activity showed a relation with higher fat free mass and better fitness in normal weight Swedish preschoolers (<10% with overweight/obesity) [23]. However, evidence is still needed for these effects, especially among at-risk and populations with overweight [21]. Moreover, studies that examine health effects in children with elevated risk status based on adiposity is a priority area identified by the 2018 Physical Activity Guidelines Scientific Advisory Committee [24]. While studies have found improved cardiovascular indicators and HRQoL when replacing sedentary time with light intensity activity or moderate to vigorous intensity physical activity (MVPA) in adults, few studies have been conducted among youth or young children [20]. Given the recent focus (e.g. whole day guidelines) that all intensity categories of movement behaviors (light-, moderate-, vigorous-intensity) are beneficial for health, examining associations in young children with elevated cardiometabolic and adiposity risk is needed.

Few time-use analyses (e.g., isotemporal, compositional) have been conducted in preschool-aged youth [23, 25–28], with only one study by Collings et al. (2017) [26] examining race/ethnicity within these relations. Therefore, the purpose of this paper is to examine the health outcomes related to the activity behaviors among a majority Hispanic sample of preschool-aged children with overweight and obesity. The study aims are to examine time replacement scenarios of movement behaviors on 1) adiposity health indicators, 2) cardiovascular health indicators, and 3) parent-reported health-related quality of life in ethnically diverse young children with overweight and obesity.

## Materials and methods

### Study design

The study is a cross-sectional analysis of baseline data of preschool-aged participants (2–5 years of age) from the Texas Childhood Obesity Research Demonstration (TX CORD) secondary prevention study. Briefly, the TX CORD secondary prevention study was a 12-month randomized controlled trial (RCT) to address childhood obesity in which children, ages 2–12 years and ≥ 85th percentile for body mass index (BMI), and their families were randomly assigned to either 1) a community-centered program or 2) a primary health care-centered program [29, 30]. Exclusion criteria for TX CORD included: 1) complications of obesity that would interfere with participation (e.g. severe respiratory insufficiency or orthopedic problems); 2) underlying obesity-related conditions, (e.g. systemic steroid use or endocrine abnormalities); 3) severe psychological problems; 4) severe obesity (>99.5th BMI percentile); and 5) participation in an obesity treatment program within the past year. In order to remove the effect of the intervention arms on the targeted health outcomes, the current study included baseline assessments only. Baseline data collection occurred between September 2012 and February 2014. At least one parent/ primary caretaker provided informed consent covering both parent and child participation. The institutional review boards at The University of Texas Health Science Center at Houston and Baylor College of Medicine approved the study protocol (HSC-SPH-11-0513).

### Accelerometer-derived movement behaviors

Physical activity was measured using ActiGraph GT3X+ devices (*ActiGraph*, *LLC*, *Pensacola*, *FL*, *USA*). Participants were asked to wear the accelerometer on their right hip (via elastic belt) for 24 hours a day for 7 consecutive days. Accelerometer files were downloaded using 15-second epoch lengths. Waking periods (06:00–22:59) were defined by examining mean hourly activity estimates [31] and then screened for non-wear periods using 20 minutes of consecutive zero counts [32, 33]. Valid wear time was set at ≥10 hours of data on ≥3 days [34, 35]. Any three days has been found to be a sufficient and reliable estimate of habitual physical activity in this age group (ICC = 0.7–0.9) [34, 36, 37]. Butte vector magnitude (VM) preschool accelerometer count cut point thresholds (2013) [38], multiplied by a factor of 0.25 (i.e., 15s/60s) [39], were used to estimate activity intensity. Averaged daily estimates (minutes per day) for 1) stationary time, 2) light intensity activity, 3) moderate intensity activity, 4) vigorous intensity activity, 5) total accumulated MVPA, and 6) total physical activity (light + MVPA) (TPA) were calculated. ‘Stationary time’ as the terminology for ‘sedentary behavior’ is in cooperation with the consensus definition provided by the Sedentary Behaviour Research Network (SBRN) that defines stationary time as sedentary behavior collected from an accelerometer that does not measure posture or context [40].

### Health indicator measures

#### Adiposity indicators

Adiposity measures included BMI z-score, the percentage of the 95th percentile (%BMIp95), fat mass, fat mass index (FMI), waist circumference, and waist to height ratio. Briefly, research staff measured height with a stadiometer and weight with a digital scale in light clothes and without shoes. Weight was measured to the nearest 0.1 kg and height was measured to the nearest 0.1 cm. Each participant was measured twice with an optional third measurement if the two measurements differed by 0.1 kg/cm. BMI z-score was computed using A SAS Program for the 2000 CDC Growth Charts and the percentage of the 95th percentile (%BMIp95) was calculated as 100 × BMI / BMI 95th percentile for age and sex [41].

Fat mass was measured using bioelectrical impedance analysis (BIA) (*TBF- 410GS*, *Tanita*, *Arlington Heights*, *Illinois*), measured to the nearest 0.1 kg. Fat mass was measured twice with the final variable equating to the average score between the two trials. Fat mass percent was calculated as fat mass (kg) / total body weight (kg). Fat mass index (FMI) was calculated as total fat mass (kg) / height^2^ (m^2^).

Waist circumference was measured to the nearest 1.0 mm using a measurement tape. Waist circumference was measured twice, in light clothes, with the final variable equating to the average score between the two trials. Total waist circumference is provided on a continuous scale (mm). Additionally, a waist to height ratio was calculated as a proportion, from 0–1.

#### Cardiovascular health indicators

Resting systolic blood pressure (SBP) and diastolic blood pressure (DBP) were measured using an automated monitor to the nearest 1.0 mmHg (8100T, Dinamap, Berlin, Massachusetts). Children were instructed to sit quietly for five minutes prior to measurement. During measurement, children were seated with their feet flat the floor. Resting SBP and DBP were measured twice with the summary variables equating to the average score between the two trials.

Resting heart rate was measured at the same time as BP using the automated monitor. The measurements were recorded to the nearest beat per minute (bpm). The final variable equates to the average score between the two trials.

#### Health-related quality of life

Health-related quality of life (HRQoL) was measured using the Pediatric Quality of Life Inventory (PedsQL), a 23-item multidimensional scale, measuring 1) Physical functioning (8 items), 2) Emotional functioning (5 items), 3) Social functioning (5 items), and 4) School functioning (5 items). Two items were removed from the School functioning sub-scale for children ages 2–5, for a final survey of 21-items. For children ages 2–5, HRQoL was measured via parent proxy-report, which has been shown to have good reliability (Cronbach’s α = 0.90) [42]. Each item has a 5-point response scale of (0 = never a problem; 1 = almost never a problem; 2 = sometimes a problem; 3 = often a problem; 4 = almost always a problem). Items were transformed to a 0–100 scale using (0 = 100, 1 = 75, 2 = 50, 3 = 25, 4 = 0) and divided by the number of items answered for each sub-scale, with higher scores indicating better HRQoL.

### Sociodemographic information

Parents were asked to complete questionnaires which included, but not limited to, questions regarding 1) sociodemographic factors of the parent, 2) demographics of the participating child, 3) parent health condition, 4) child strengths and difficulties questionnaire, 5) child well-being, and 6) child’s growth.

### Data analysis

Secondary analyses were conducted in July 2020. Participants in the 2–5 age group, who returned the accelerometer, had completed parent surveys, and completed health measures (N = 141) were included. Of these, only preschool-aged children with valid accelerometer wear time of ≥10 hours of data between the hours of 06:00–22:59 on ≥3 days were included in the analytic sample (N = 131). Statistical analyses were performed using SAS 9.4 (*SAS Institute Inc*., *Cary*, *NC*). Descriptive statistics were conducted for demographic, physical activity variables, and health indicators and stratified by sex. Variables were assessed for normality both visually and through the Shapiro-Wilk test, with medians and interquartile ranges presented for non-normal data and means and standard deviations provided for normally distributed data. Mann-Whitney U test or t-tests, depending on normality, compared differences across sex at α = 0.05. Outliers were examined for each physical activity variables and health indicators.

The isotemporal substitution modeling approach [19] was used to examine the hypothetical effect on health when substituting movement behaviors using multiple linear regression models. Prior to entering the movement variables into the models, movement variables were divided by 5. This was used so that a one-unit change was equitable to 5 minutes per day. Covariates included age (years), sex (male/female), ethnicity (Hispanic/ non-Hispanic), and socioeconomic status (income to poverty ratio). Additionally, as associations between physical activity and adiposity have been found to differ for boys and girls [43], a subanalyses examined potential differences in adiposity changes by sex. Two outliers, defined as an observation 3 times above or below the interquartile range, were removed from the isotemporal substitution models.

## Results

The demographic, health indicators, and accelerometer-derived movement behaviors are presented in **Table 1**. The analytic sample had a mean age of 4.3 (SD 1.1), was 52% female, 87% Hispanic, and a median income to poverty ratio of 62.6%. The sample had a median 109% (IQR: 100–123) of the 95th percentile for BMI, high ratings for overall and physical functioning HRQoL, and had a mean SBP of 95.2 (SD 9.6) mmHg and 59.5 (SD 7.4) DBP mmHg, which equated to 13.2% of the sample having elevated SBP or worse (≥90th percentile) and 26.5% having at least elevated DBP (≥90th percentile), based on age, sex, and height determined guidelines [44].

**Table 1:** Characteristics of the preschool-aged participants.

Characteristics	Overall (N = 131)		Boys (n = 62)		Girls (n = 69)		p-value
Age (y), M ± SD	4.3 ± 1.1		4.4 ± 1.0		4.3 ± 1.2		0.455
Female, % (n)	52.7	(69)					
Hispanic, % (n)	87.0	(114)	83.9	(52)	89.9	(62)	0.436
Income to Poverty Ratio (%), Median (IQR)	62.6	(45.0, 93.4)	64.8	(50.2, 95.2)	53.3	(42.3, 80.9)	0.064
Adiposity Indicators, Median (IQR)							
BMI z score (kg/m^2^)	2.2	(1.7, 2.9)	2.1	(1.7, 3.1)	2.3	(1.9, 2.7)	0.894
%BMIp95 (%)	108.7	(100.5, 122.6)	105.9	(100.2, 122.1)	111.9	(103.4, 122.6)	0.231
Fat mass %	31.7	(27.4, 36.1)	28.5	(25.7, 34.5)	32.8	(29.2, 37.0)	**0.003**
Fat mass index (kg/m^2^)	6.2	(5.0, 8.2)	5.4	(4.7, 7.8)	6.6	(5.5, 8.3)	**0.006**
Waist circumference (cm)	62.5	(56.3, 69.0)	59.2	(55.0, 67.4)	63.0	(57.3, 70.1)	0.099
WHtR	0.58	(0.54, 0.64)	0.56	(0.54, 0.61)	0.59	(0.56, 0.65)	**0.001**
Cardiovascular Indicators, M ± SD							
Resting systolic blood pressure (mmHg)	95.2 ± 9.6		96.6 ± 9.2		94.0 ± 9.9		0.142
Resting diastolic blood pressure (mmHg)	59.5 ± 7.4		59.2 ± 7.6		59.2 ± 7.3		0.682
Resting heart rate (bpm)	94.9 ± 13.1		91.8 ± 11.8		97.6 ± 13.6		**0.015**
Health-related Quality of Life Indicators, Median (IQR)							
Overall HRQoL	90.5	(80.4, 96.4)	90.5	(79.8, 95.2)	91.7	(83.3, 97.6)	0.336
Physical functioning HRQoL	95.3	(85.7, 100.0)	93.8	(85.7, 100.0)	96.9	(81.3, 100.0)	0.544
Accelerometer-derived Movement Behaviors (min/d), Median (IQR)							
Accelerometer wear time	865.3	(818.4, 895.3)	872.1	(834.0, 899.5)	860.3	(812.1, 891.9)	0.153
Stationary time	469.8	(424.3, 519.0)	476.2	(421.7, 509.0)	461.6	(427.9, 523.8)	0.770
Light intensity activity	316.9	(284.4, 347.4)	311.1	(274.7, 345.3)	322.6	(301.7, 347.4)	0.394
Moderate intensity activity	55.3	(42.3, 67.6)	62.5	(52.3, 69.9)	47.8	(37.7, 58.7)	**<0.001**
Vigorous intensity activity	15.8	(10.4, 23.0)	20.0	(15.2, 28.2)	12.2	(9.5, 18.3)	**<0.001**
Total MVPA	71.9	(54.0, 90.3)	82.8	(64.8, 100.6)	61.8	(45.5, 76.7)	**<0.001**
Total physical activity (TPA)	393.5	(350.0, 432.7)	409.5	(351.3, 445.2)	383.3	(350.0, 415.8)	0.140
Average vector magnitude counts (ct×min/d)	1174.5	(1033.7, 1291.7)	1252.4	(1118.3, 1321.1)	1126.7	(953.0, 1260.9)	**0.004**
Calendar days	7.0	(6.0, 7.0)	7.0	(6.0, 7.0)	7.0	(6.0, 7.0)	0.154

Abbreviations: BMI, body mass index; ct, count; d, day; FMI, fat mass index; HRQoL, Health-related quality of life; IQR, interquartile range; M, mean; min, minutes; MVPA, moderate to vigorous physical activity; SD, standard deviation; TPA, total physical activity; WHtR, Waist to Height Ratio; y, years; %BMIp95, percentage of the 95th percentile.

Isotemporal substitution models for each health indicator were run separately. Isotemporal substitution models for adiposity health indicators **(Table 2)**, cardiovascular health indicators **(Table 3)**, and HRQoL (**Table 4)** for the overall sample are presented. Adiposity models by sex, males (n = 60) and female participants (n = 69), are presented in **Table 5**. In addition to age, sex, ethnicity, and socioeconomic status, the cardiovascular health models controlled for child height. The male and female adiposity models removed sex as a covariate, controlling for age (years), ethnicity (Hispanic/ non-Hispanic), and socioeconomic status (income to poverty ratio). Sex-stratified isotemporal substitution models were only significant for males.

**Table 2:** Isotemporal substitution of movement behaviors with adiposity health indicators in preschool children with overweight and obesity participating in the baseline assessment of the TX CORD secondary prevention study (N = 131).

	BMI z-score	%BMIp95	Fat mass (%)	FMI (kg/m^2^)	Waist circumference (cm)	Waist to height ratio
	β (95% CI) (n = 128)	β (95% CI) (n = 128)	β (95% CI) (n = 121)	β (95% CI) (n = 121)	β (95% CI) (n = 126)	β (95% CI) (n = 126)
Isotemporal Individual Intensity Models						
LPA → Stationary	**-0.02 (-0.04, -0.01)**	-0.24 (-0.60, 0.12)	-0.09 (0.23, 0.05)	-0.03 (-0.08, 0.03)	-0.09 (-0.28, 0.11)	-0.01 (-0.01, 0.01)
MPA → Stationary	**0.11 (0.03, 0.19)**	**1.64 (0.23, 3.05)**	**0.59 (0.03, 1.14)**	0.19 (-0.01, 0.40)	0.93 (-0.18, 1.70)	0.01 (-0.01, 0.01)
VPA → Stationary	**-0.13 (-0.26, -0.01)**	**-2.56 (-4.78, -0.34)**	**-1.24 (-2.01, -0.38)**	**-0.41 (-0.73, -0.09)**	**-1.75 (-2.95, -0.55)**	**-0.01 (-0.02, -0.01)**
MPA → LPA	**0.13 (0.04, 0.22)**	**1.88 (0.24, 3.51)**	**0.68 (0.03, 1.32)**	0.22 (-0.02, 0.46)	**1.02 (0.14, 1.90)**	0.01 (-0.01, 0.01)
VPA → LPA	-0.11 (-0.23, 0.01)	**-2.33 (-4.45, -0.20)**	**-1.15 (-1.98, -0.33)**	**-0.38 (-0.69, -0.08)**	**-1.67 (-2.81, -0.52)**	**-0.01 (-0.02, -0.01)**
VPA → MPA	**-0.24 (-0.43, -0.05)**	**-4.20 (-7.60, -0.80)**	**-1.83 (-3.15, -0.52)**	**-0.60 (-1.09, -0.12)**	**-2.69 (-4.52, -0.85)**	**-0.02 (-0.03, -0.01)**
Isotemporal MVPA Models						
LPA → Stationary	-0.01 (-0.03, 0.01)	-0.03 (-0.36, 0.30)	-0.01 (-0.13, 0.13)	0.01 (-0.04, 0.05)	0.04 (-0.14, 0.22)	0.01 (-0.01, 0.01)
MVPA → Stationary	0.02 (-0.01, 0.06)	0.08 (-0.55, 0.71)	-0.10 (-0.36, 0.16)	-0.03 (-0.13, 0.06)	-0.07 (-0.41, 0.28)	-0.01 (-0.01, 0.01)
MVPA → LPA	0.03 (-0.01, 0.08)	0.41 (-0.70, 0.92)	-0.10 (-0.43, 0.23)	-0.04 (-0.16, 0.08)	-0.11 (-0.55, 0.33)	-0.01 (-0.01, 0.01)
Isotemporal TPA Models						
TPA → Stationary	-0.01 (-0.02, 0.01)	-0.01 (-0.24, 0.23)	-0.03 (-0.12, 0.07)	-0.01 (-0.04, 0.03)	0.01 (-0.12, 0.14)	-0.01 (-0.01, 0.01)

Abbreviations: BMI, body mass index; CI, confidence interval; cm, centimeter; FMI, fat mass index; LPA, light intensity physical activity; MPA, moderate intensity physical activity; MVPA, moderate to vigorous physical activity; VPA, vigorous intensity physical activity; TPA, total physical activity; %BMIp95, percentage of the 95th percentile.

Note: Models are per 5-min change.

The arrow (→) indicates a time replacement scenario. For example, MVPA → Stationary indicates 5 minutes of MVPA replacing 5 minutes of stationary activity.

Model covariates: age, sex, ethnicity, and socioeconomic status.

**Table 3:** Isotemporal substitution of movement behaviors with cardiovascular health indicators in preschool children with overweight and obesity participating in the baseline assessment of the TX CORD secondary prevention study (N = 131).

	Resting Systolic BP	Resting Diastolic BP	Resting Heart Rate (bpm)
	β (95% CI) (n = 118)	β (95% CI) (n = 118)	β (95% CI) (n = 119)
Isotemporal Individual Intensity Models			
LPA → Stationary	-0.17 (-0.41, 0.09)	-0.10 (-0.29, 0.09)	0.01 (-0.31. 0.32)
MPA → Stationary	0.47 (-0.51, 1.45)	0.64 (-0.11, 1.39)	-0.34 (-1.62, 0.88)
VPA → Stationary	**-2.23 (-3.72, -0.74)**	**-2.14 (-3.28, -0.99)**	-0.89 (-2.79, 1.02)
MPA → LPA	0.64 (-0.50, 1.77)	0.75 (-0.13, 1.62)	-0.37 (-1.83, 1.08)
VPA → LPA	**-2.07 (-3.48, -0.65)**	**-2.03 (-3.12, -0.94)**	-0.89 (-2.71, 0.92)
VPA → MPA	**-2.70 (-5.00, -0.40)**	**-2.78 (-4.55, -1.00)**	-0.52 (-3.47, 2.43)
Isotemporal MVPA Models			
LPA → Stationary	-0.03 (-0.25, 0.19)	0.04 (-0.14, 0.21)	0.03 (-0.24, 0.31)
MVPA → Stationary	**-0.56 (-0.99, -0.12)**	**-0.42 (-0.76, -0.08)**	**-0.57 (-1.11, -0.02)**
MVPA → LPA	-0.53 (-1.09, 0.03)	**-0.45 (-0.89, -0.02)**	-0.60 (-1.30, 0.10)
Isotemporal TPA Models			
TPA → Stationary	**-0.17 (-0.33, -0.01)**	-0.09 (-0.21, 0.04)	-0.13 (-0.33, 0.06)

Abbreviations: BP, blood pressure; CI, confidence interval; LPA, light intensity physical activity; MPA, moderate intensity physical activity; MVPA, moderate to vigorous physical activity; TPA, total physical activity; VPA, vigorous intensity physical activity.

Note: Models are per 5-min change

The arrow (→) indicates a time replacement scenario. For example, MVPA → Stationary indicates 5 minutes of MVPA replacing 5 minutes of stationary activity.

Model covariates: age, sex, ethnicity, socioeconomic status, and child height.

**Table 4:** Isotemporal substitution of movement behaviors with health-related quality of life in preschool children with overweight and obesity participating in the baseline assessment of the TX CORD secondary prevention study (N = 131).

	Overall HRQoL	Physical Functioning HRQoL
	β (95% CI) (n = 121)	β (95% CI) (n = 121)
Isotemporal Individual Intensity Models		
LPA → Stationary	-0.10 (-0.43, 0.23)	-0.41 (-0.85, 0.03)
MPA → Stationary	0.04 (-1.22, 1.30)	0.52 (-1.16, 2.20)
VPA → Stationary	0.35 (-1.63, 2.34)	-0.52 (-3.18, 2.13)
MPA → LPA	0.14 (-1.33, 1.61)	0.93 (-1.03, 2.89)
VPA → LPA	0.45 (-1.44, 2.34)	-0.11 (-2.64, 2.41)
VPA → MPA	0.31 (-2.73, 3.36)	-1.04 (-5.12, 3.03)
Isotemporal MVPA Models		
LPA → Stationary	-0.12 (-0.41, 0.17)	-0.36 (-0.74, 0.03)
MVPA → Stationary	0.16 (-0.38, 0.69)	0.13 (-0.59, 0.85)
MVPA → LPA	0.27 (-0.42, 0.97)	0.49 (-0.44, 1.42)
Isotemporal TPA Models		
TPA → Stationary	-0.04 (-0.25, 0.17)	-0.22 (-0.49, 0.06)

Abbreviations: CI, confidence interval; HRQoL, health-related quality of life; LPA, light intensity physical activity; MPA, moderate intensity physical activity; MVPA, moderate to vigorous physical activity; TPA, total physical activity; VPA, vigorous intensity physical activity.

Note: Models are per 5-min change

The arrow (→) indicates a time replacement scenario. For example, MVPA → Stationary indicates 5 minutes of MVPA replacing 5 minutes of stationary activity.

Model covariates: age, sex, ethnicity, and socioeconomic status

**Table 5:** Isotemporal substitution of movement behaviors with adiposity health indicators by sex in preschool children with overweight and obesity participating in the baseline assessment of the TX CORD secondary prevention study (N = 131).

	BMI z-score	%BMIp95	Fat mass (%)	FMI (kg/m^2^)	Waist circumference (cm)	Waist to height ratio
	β (95% CI)	β (95% CI)	β (95% CI)	β (95% CI)	β (95% CI)	β (95% CI)
**Male Only (n = 60)**						
Isotemporal Individual Intensity Models						
LPA → Stationary	**-0.05 (-0.08, -0.02)**	**-0.73 (-1.21, -0.25)**	**-0.24 (-0.46, -0.01)**	**-0.08 (-0.16, -0.01)**	**-0.34 (-0.60, -0.07)**	**-0.01 (-0.01, -0.01)**
MPA → Stationary	**0.17 (0.03, 0.31)**	**2.33 (0.16, 4.50)**	0.74 (-0.30, 1.78)	0.25 (-0.11, 0.60)	**1.29 (0.07, 2.51)**	0.01 (-0.01, 0.01)
VPA → Stationary	-0.18 (-0.37, 0.01)	**-3.10 (-6.10, -0.10)**	**-1.51 (-2.86, -0.15)**	**-0.48 (-0.94, -0.02)**	**-1.97 (-3.65, -0.30)**	-0.01 (-0.02, 0.01)
MPA → LPA	**0.22 (0.06, 0.37)**	**3.06 (0.62, 5.51)**	0.98 (-0.20, 2.15)	0.33 (-0.07, 0.74)	**1.63 (0.26, 3.00)**	0.01 (-0.01, 0.02)
VPA → LPA	-0.13 (-0.31, 0.05)	-2.37 (-5.36, 0.52)	-1.27 (-2.57, 0.03)	-0.39 (-0.84, 0.05)	**-1.64 (-3.25, -0.02)**	-0.01 (-0.02, 0.01)
VPA → MPA	**-0.35 (-0.65, -0.04)**	**-5.43 (-10.30, -0.57)**	**-2.25 (-4.47, -0.02)**	-0.73 (-1.49, 0.04)	**-3.26 (-5.99, -0.54)**	-0.02 (-0.04, 0.01)
Isotemporal MVPA Models						
LPA → Stationary	**-0.03 (-0.06, -0.01)**	**-0.51 (-0.96, -0.06)**	-0.14 (-0.35, 0.07)	-0.05 (-0.12, 0.02)	-0.20 (-0.46, 0.05)	-0.01 (-0.01, 0.01)
MVPA → Stationary	0.03 (-0.03, 0.08)	0.11 (-0.78, 1.00)	-0.21 (-0.66, 0.23)	-0.06 (-0.21, 0.09)	-0.05 (-0.55, 0.45)	-0.01 (-0.01, 0.01)
MVPA → LPA	0.06 (-0.01, 0.13)	0.62 (-0.51, 1.75)	-0.08 (-0.64, 0.49)	-0.01 (-0.20, 0.18)	0.16 (-0.48, 0.79)	0.01 (-0.01, 0.01)
Isotemporal TPA Models						
TPA → Stationary	-0.02 (-0.04, 0.01)	**-0.35 (-0.68, -0.01)**	**-0.16 (-0.31, -0.01)**	**-0.05 (0.11, -0.01)**	-0.16 (-0.35, 0.02)	-0.01 (-0.01, 0.01)
**Female Only (n = 69)**						
Isotemporal Individual Intensity Models						
LPA → Stationary	0.01 (-0.02, 0.04)	0.41 (-0.14, 0.96)	0.11 (-0.08, 0.30)	0.05 (-0.03, 0.13)	0.24 (-0.05, 0.53)	0.01 (-0.01, 0.01)
MPA → Stationary	0.03 (-0.06, 0.12)	0.19 (-1.72, 2.09)	0.13 (-0.52, 0.78)	0.03 (-0.23, 0.29)	0.19 (-0.82, 1.20)	0.01 (-0.01, 0.01)
VPA → Stationary	-0.07 (-0.22, 0.09)	-1.01 (-4.38, 2.35)	-0.55 (-1.71, 0.60)	-0.18 (-0.64, 0.28)	-1.01 (-2.79, 0.78)	-0.01 (-0.02, 0.01)
MPA → LPA	0.02 (-0.09, 0.12)	-0.22 (-2.51, 2.06)	0.02 (-0.76, 0.80)	-0.02 (-0.34, 0.29)	-0.05 (-1.25, 1.16)	0.01 (-0.01, 0.01)
VPA → LPA	-0.08 (-0.23, 0.08)	-1.42 (-4.62, 1.77)	-0.66 (-1.76, 0.43)	-0.23 (-0.67, 0.21)	-1.24 (-2.94, 0.45)	-0.01 (-0.02, 0.01)
VPA → MPA	-0.09 (-0.32, 0.14)	-1.20 (-6.13, 3.73)	-0.68 (-2.38, 1.01)	-0.21 (-0.88, 0.47)	-1.20 (-3.81, 1.42)	-0.01 (-0.03, 0.01)
Isotemporal MVPA Models						
LPA → Stationary	0.02 (-0.01, 0.04)	**0.48 (0.03, 0.95)**	0.15 (-0.02, 0.31)	0.06 (-0.01, 0.13)	**0.30 (0.05, 0.55)**	0.01 (-0.01, 0.01)
MVPA → Stationary	-0.01 (-0.05, 0.04)	-0.22 (-1.11, 0.66)	-0.10 (-0.41, 0.20)	-0.04 (-0.16, 0.08)	-0.22 (-0.69, 0.25)	-0.01 (-0.01, 0.01)
MVPA → LPA	-0.02 (-0.08. 0.03)	-0.70 (-1.87, 0.46)	-0.25 (-0.65, 0.15)	-0.10 (-0.26, 0.05)	-0.52 (-1.14, 0.10)	-0.01 (-0.01, 0.01)
Isotemporal TPA Models						
TPA → Stationary	0.01 (-0.01, 0.02)	0.27 (-0.06, 0.60)	0.07 (-0.04, 0.19)	0.03 (-0.02, 0.08)	0.15 (-0.03, 0.32)	0.01 (-0.01, 0.01)

Abbreviations: BMI, body mass index; CI, confidence interval; cm, centimeter; FMI, fat mass index; LPA, light intensity physical activity; MPA, moderate intensity physical activity; MVPA, moderate to vigorous physical activity; TPA, total physical activity; VPA, vigorous intensity physical activity; %BMIp95, percentage of the 95th percentile.

Note: Models are per 5-min change

The arrow (→) indicates a time replacement scenario. For example, MVPA → Stationary indicates 5 minutes of MVPA replacing 5 minutes of stationary activity.

Model covariates: age, ethnicity, and socioeconomic status.

### Replacing stationary behavior

Replacing 5 minutes of stationary time with 5 minutes of light intensity activity showed a relation with a reduction in BMI z-score (-0.02). Replacing stationary time with moderate intensity physical activity showed a relation with increased BMI z-score (0.11), percentage of the 95th percentile (1.6%), and percent fat mass (0.6%). Replacing stationary time with vigorous intensity physical activity showed a relation with reductions in BMI z-score (-0.13), percentage of the 95th percentile (-2.6%), fat mass percent (-1.2%), FMI (-0.4 kg/m^2^), waist circumference (-1.8 cm), waist to height ratio (-0.01), resting SBP (-2.7 mmHg), and resting DBP (2.7 mmHg). In the accumulated MVPA models, replacing 5 minutes of stationary time with 5 minutes of MVPA showed a relation with a 0.6 mmHg decrease in resting SBP, 0.4 mmHg decrease in resting DBP, and 0.6 bpm reduction in resting heart rate. In the accumulated TPA models, replacing 5 minutes of stationary time with 5 minutes of TPA showed a relation with a 0.2 mmHg decrease in resting systolic BP. No significant reallocating time scenarios were found for HRQoL.

In the sex stratified adiposity models, models were only significant for boys. Additionally, replacing stationary time with light intensity physical activity showed a relation with reductions in BMI z-score (0.05) and percentage of the 95th percentile (0.7%) in boys. Further, replacing stationary behavior with total physical activity showed a relation with reductions in percentage of the 95th percentile (0.4%), fat mass (0.2%), and fat mass index (0.1 kg/m^2^) in boys. In contrast, in girls, replacing stationary behavior with light intensity showed a relation with an increase in percentage of the 95th percentile (0.5%) and waist circumference (0.3 cm).

### Replacing light intensity

Replacing light intensity with moderate intensity physical activity showed a relation with increases in BMI z-score (0.1), percentage of the 95th percentile (1.9%), fat mass percentage (0.7%), and waist circumference (1.0 cm). Replacing 5 minutes of light intensity with 5 minutes of vigorous intensity activity showed a relation with reductions in percentage of the 95th percentile (-2.3%), fat mass percent (-1.2%), FMI (-0.4 kg/m^2^), waist circumference (-1.7 cm), waist to height ratio (-0.01), resting SBP (-2.0 mmHg), and resting DBP (-2.0 mmHg). In the accumulated MVPA model, replacing 5 minutes of light intensity activity with 5 minutes of MVPA showed a relation with a 0.5 mmHg reduction in DBP. The sex stratified adiposity models were only significant for boys. No significant reallocating time scenarios were found for HRQoL.

### Replacing moderate intensity

Replacing 5 minutes of moderate intensity with 5 minutes of vigorous intensity activity showed a relation with reductions in BMI z-score (-0.2), percentage of the 95th percentile (-4.2%), fat mass percent (-1.8%), FMI (-0.6 kg/m^2^), waist circumference (-2.7 cm), waist to height ratio (-0.02), resting SBP (-2.7 mmHg), and resting DBP (-2.8 mmHg). The sex stratified adiposity models were only significant for boys. No significant reallocating time scenarios were found for HRQoL.

## Discussion

This study examined adiposity, cardiovascular, and health-related quality of life outcomes related to movement behaviors among primarily Hispanic preschool children with overweight and obesity in Texas. The principal finding of this paper is that reallocation of time spent stationary, in light, or moderate intensity physical activity with vigorous intensity physical activity is associated with several favorable adiposity and cardiovascular health outcomes among preschool-aged children with overweight and obesity. High intensity exercise training has been found to be effective stimulus in reducing body composition and abdominal fat in adults–which may be due to induced enzyme activity [45, 46]. This study suggests that higher intensity activity may have similar effects in young children.

To our knowledge, only one previous study has been conducted in a Hispanic preschool population with clinical overweight, which found that greater accelerometer-determined MVPA was associated with lower BMI z-score [47]. This study further builds upon the study by Mendoza et al. (2014) by showing higher intensity exercise lowers a variety of obesity indicators. The benefits to health when replacing stationary time and lower intensity activities with vigorous intensity physical activity found in this study are supported by previous literature that found associations between MVPA and adiposity were weaker than associations for vigorous intensity, which may suggest adiposity in young children is more strongly associated with vigorous intensity activity [23, 48]. However, given these data are cross-sectional, only hypothetical time replacement scenarios on outcomes can be examined. Further exploration, particularly with more rigorous research designs such as prospective or longitudinal studies, are needed. While this study supports previous findings that substituting five minutes per day of stationary time, light, or moderate intensity activity with vigorous intensity activity shows relations with better health indicators [23], the findings of this study may further suggest that these associations between vigorous intensity physical activity and body composition appear stronger within an overweight population. As most studies to date have been conducted primarily among populations with low overweight/obesity percentages (8.5%– 20%) [23, 26], understanding the unique contributions of vigorous intensity among preschoolers with overweight is warranted.

The direct association between adiposity indicators and moderate intensity physical activity was not expected. Further exploring this relation by sex, the direct association between adiposity indicators was significant only for boys. Previous studies have found a significant interaction by sex when examining prolonged sitting time with waist circumference and clustered cardiometabolic risk among older children (Mean age = 11.2±2.7) [22]. Although outliers for both health and physical activity were removed, males in this study had greater variability around the median value and significantly higher daily minutes of MVPA compared to females, which may have contributed to the differing associations between the sexes. Further examining hourly patterns by sex may provide a potential mechanism as physical activity can regulate energy metabolism, sensitivity, and regulation of body homeostasis [49]. Other explanations for these findings are the potential misclassification of activity thresholds when 1) using accelerometer cut points validated in normal weight populations in populations with overweight and 2) using individual intensity classifications in this population. Findings from Raiber et al. (2018) suggest relative intensity cut point thresholds for individuals with overweight and obesity are lower than individuals with normal weight [50]. Thus, some stationary time may be misrepresented as light intensity activity; light intensity activity as moderate intensity; and moderate intensity as vigorous intensity. Additionally, classification rates for Butte VM cut point thresholds are 83, 64, 35, 38% for sedentary, light, moderate and vigorous levels of physical activity, however when collapsing moderate and vigorous intensity activity into a single MVPA category, overall accuracy increases to 83, 64, 63% [38]. Given this, the individual intensity models should be interpreted with caution. Future research needs to examine these findings using cut points developed specially for young children with overweight and the examination of hourly activity patterns, such as diurnal patterns, impact on health in this population.

This is one of the first studies to examine isotemporal substitution models regarding cardiovascular indicators among preschool-aged children. Although studies have found improved cardiovascular indicators when replacing stationary time with light intensity activity or MVPA in adults [20] and more recently children [22], no studies have been conducted among only preschool-aged children. Research regarding BP in young children is of the utmost importance as the American Academy of Pediatrics (AAP) recommends that BP is measured annually in children aged three and older and checked at every health care encounter if they have obesity [44]. This study found that among preschool-aged children with overweight, vigorous intensity physical activity is inversely associated with SBP. Substituting 5 minutes per day of vigorous intensity activity for stationary time, light, and moderate intensity activity showed a relation with a 2 mmHg reduction in SBP. This is important as 13.2% of the sample had elevated or higher SBP. In the AAP pediatric hypertension guidelines [44], about 5 mmHg separates categorization from elevated BP (≥90^th^ percentile) to stage 1 hypertension (≥95^th^ percentile), and about 15 mmHg separates categorization from elevated BP to stage 2 hypertension (≥95^th^ + 12 mmHg), in preschool-aged children. Therefore, clinical relevance may only occur with larger time replacements, such as 10 to 40-minute reductions in stationary time. Reducing elevated BP is of importance as elevated BP tracks from childhood into adulthood [44, 51, 52]. Promoting vigorous intensity physical activity during the early childhood years can be important for managing elevated BP levels and, conceivably, reducing the risk of hypertension and heart disease into adulthood.

In regards to quality of life, parents with preschoolers who have overweight and obesity have been found to rate their child’s walking, running, ability to lift something heavy, and energy level as lower than parents of normal weight children [10]. Further, compared to non-overweight children, children with clinical obesity are 72% more likely to have additional health care needs [11]. This is the first study to use isotemporal substitution models to assess changes in HRQoL when hypothetical substitutions of movement behaviors occur within this population. While studies have found improved HRQoL when replacing stationary time with physical activity in adults [20] and although this sample has poor adiposity and cardiovascular health indicators, the relatively high scores for overall HRQoL (Mean = 90.5) and physical functioning (Mean = 95.3) may have contributed to the lack of association between physical activity and quality of life within this study. Future studies should continue to examine the association between physical activity and HRQoL and physical functioning in more diverse populations (e.g. race/ethnicity, BMI status).

### Strengths and limitations

This study has a number of strengths including use of a clinical and ethnically diverse population. Hispanic youth have highest prevalence of overweight (29.8%) and obesity (15.6%) compared to other race/ethnic groups in the U.S. [16, 17], yet few studies have examined the relation between movement behaviors and health indicators in Hispanic youth experiencing obesity. The use of a device-based measure of physical activity and age-specific VM cut-points, allows for the assessment of specific intensity minutes. This is especially important as the whole day guidelines include light intensity physical activity, which is not easily captured via self- or parent-proxy report measures. Further, the use of isotemporal data analysis considers the nature of time and multicollinearity (e.g., high correlation between estimates of sedentary time and light intensity physical activity)–a limitation in previous studies that examine independent associations of physical activity and health.

This is the first study to use isotemporal analyses with cardiovascular indicators and HRQoL in preschool-aged youth. Previous studies examining movement behavior combinations have primarily focused on motor development, fitness, and favorable adiposity outcomes among this age group [53]. By examining movement behaviors and HRQoL, this study addresses a key gap in the literature. Additionally, this is one of the first studies to examine the relation between physical activity and BP using a device-based physical activity measurement. Further strengths include use of bioelectrical impedance, as a major limitation of the current literature is the use of BMI as only of the only indicators of adiposity, especially as BMI may not be the best indicator of adiposity in growing children [54].

Limitations should be noted. This is a cross-sectional study, so it is unable to determine causality or analyze how this relation affects health over time. Further, while the isotemporal data model approach allows for the evaluation of the “impact” of counterfactual time replacement scenarios–an important step for research–it should be noted there are other statistical methods for time-use data (e.g., compositional data analyses). Given that compositional and non-compositional methods have been found to produce broadly similar results [55, 56], the large body of physical activity epidemiology research using isotemporal substitutions methods allowing for comparison of findings, and the ease of interpretation of absolute values (i.e., min/day) [55], the isotemporal data model approach was used. However, consequently, the findings of this study may only be directly comparable to other studies using the Mekary et al. [19] approach given the differences in the variety of statistical approaches [20]. More research is needed about the appropriateness between isotemporal and compositional analyses in physical activity research.

Misclassification of movement thresholds when using devices is always a concern. In this study, the use of VM cut point thresholds may have categorized children as more active and less time spent stationary than use of vertical axis cut points [57]. This study was also limited to cut points derived for waist placement. Wrist-worn devices have become increasingly popular and may aid in sleep detection [33] enabling researchers to more accurately measure 24-hour movement and non-movement behaviors. Additionally, as noted previously, cut points specifically designed for preschool-aged children with overweight or obesity are needed as intensity cut point thresholds may be lower for those experiencing obesity [50]. Future research should derive cut point thresholds for waist- and wrist-worn devices for use in overweight populations. Despite the good overall classification accuracy of the cut points used in this study [38], it may be that utilization of different propriety counts would yield different results. Further, as noted by Butte and colleagues [38], activity intensity classification accuracy may be best when collapsing MVPA into a single category. Given these potentials for intensity misclassification, the individual intensity models should be interpreted with caution.

Additional limitations include the lack of gold-standard measure of adiposity, such as dual-energy X-ray absorptiometry (DXA) and accuracy of the blood pressure device. Despite potential overestimation of blood pressure, the device has been recommended for use in epidemiological surveys in children [58]. While bioelectrical impedance is an improvement over only using BMI for measuring adiposity, there is still error with using this measure for fat mass. Additionally, participants were not provided instructions (e.g., requirement to fast) prior to entering the clinic for the health assessments, which may have reduced the accuracy of the health assessment results. For example, for the most accurate BIA results, participants should have fasted for four hours and drank at least one quart of water one hour prior to testing. Additionally, there may have been ceiling effects for HRQoL as scores were generally high, which may limit generalizability of this study. Finally, while this population is of importance, generalizability is limited to low-income, primarily Hispanic preschool youth with obesity.

## Conclusions

The study is one of the first studies to use isotemporal substitution modeling to examine the impact of movement behaviors on a variety of health-related indicators in a sample of majority Hispanic preschoolers with overweight and obesity. This study suggests that vigorous intensity physical activity types should be encouraged for health benefits among preschoolers. As school and home are two of the most important settings for children [59], teaching caregivers (parents and teachers) how to engage children in vigorous intensity is needed, especially since children with overweight spend more time sedentary and less time in higher intensity physical activities than normal weight children [28, 60]. Additionally, the results of this study can help researchers and clinicians develop interventions and counsel families on ways to promote healthy growth during this important stage in development.
